# On Mineral Waters

**Published:** 1856-05

**Authors:** 


### ECLECTIC DEPARTMENT,

### AND SPIRIT OF THE MEDICAL PERIODICAL PRESS

*On Mineral Waters.* By S. Hanbury Smith, M. D., of Hamilton, Ohio

As already observed, the *matters dissolved in mineral waters*, consist of most of those haloid, metallic, alkaline and earthy salts which are daily prescribed by the physician, thus:

*Sodium* is found as the chloride, iodide and bromide, and in the sulphate and carbonate of soda.

*Calcium* as the chloride, and in the carbonate, sulphate, phosphate, sub-phosphate, and fluate of lime.

*Magnesium* as the chloride, and in the sulphate, nitrate and carbonate of magnesia.

*Iron* as the carbonate, sulphate, and crenate, generally of the protoxide.

*Potassium* as the sulphate, carbonate and acetate of potassa.

*Alluminium* as the sulphate, and sub-phosphate of alumina, and as ammonia, potassa, and soda-alum.

*Manganese* as the carbonate, and sulphate of the protoxide.

*Zinc* as the sulphate.

Other mineralizing ingredients are found so rarely, in such small quantities, or are medicinally so indifferent, that it is not worth while to particularize the forms in which they occur.

The *gases* found in mineral waters, and some of which play a very important part as therapeutic agents, are carbonic acid, hydro-sulphuric acid, nitrogen and oxygen.

It would be entirely out of place in these short articles, to repeat that which is to be found in every text-book of Materia Medica, concerning the properties and *modus operandi* of the agents, just enumerated. The cause of the different, in many cases superior medical effects produced by the same agents when employed as "mineral waters," is what concerns us. I think the reflecting reader will be prepared to understand and admit, that the solution of the active remedies in such large proportions of water, hot or cold, is the most important condition of increased curative energy and advantageously modified action. The amount of water taken with almost any medicine materially affects its operation, a circumstance much too little thought of in daily prescription. To exert a general or what may be termed a remote action on the system, every medicine must be absorbed. I should indeed, perhaps, have commenced by remarking on the extreme difficulty with which it can even gain admission into the body, unless actually dissolved by the animal fluids; and it by no means follows that it is best to administer medicines by the mouth in order to ensure such true access to the organism, though our lamentable ignorance of the principles which should guide the practitioner in his choice of the best modes of exhibiting his remedies, has undoubtedly shut us out from a large portion of the field in which therapeutic operations should be carried on.

Mialhe's treatise on the art of prescribing, etc., has lifted a corner of the veil, and given us a glimpse of tempting unexplored regions for scientific research; but the whole subject of endermic medication — the *iatra leptic* method—sadly needs its Liebig and Pereira. Thus many a medicine applied to the skin seems inert merely because we do not know how, in what shape or solution best to apply the remedy. The secretion from a blistered surface, for example, being alkaline and very like that poured out on the surface of the intestines, a proper knowledge of the composition and chemical reactions of any medicine will at once decide whether or not it can be absorbed by the abraded skin. The digestive canal owes its superiority as a locality from whence insoluble medicines may find admission into the system, mainly to the circumstance, that there are "acids in the stomach, alkalies in the intestines, and saline matters in every part of the canal." In mineral waters, every medicinal ingredient is already dissolved, and that in such large quantities of the solvent, that it readily finds its way to every part of the system, when not debarred at its very entrance by decomposition; these solutions are indeed often more digestible, as it is termed, really more rapidly absorbed, than the same amount of pure water; a circumstance probably due to their exciting vital energy and to the influence of endosmosis.

Space will not allow of more than a few suggestive hints as to the therapeutic action of the chief ingredients of mineral waters.

Thus the purgative neutral salts which occur so frequently in these combinations of dame Nature's prescribing act more gently and yet more thoroughly, with much less perturbation in proportion to the effects produced, than equivalent or even larger doses of Glauber, Epsom, or Rochelle. Two ounces of Püllna water containing not more than 50 grains of principally sulphate of soda, carbonate and muriate of magnesia, and sulphate of potassa, will more effectually cleanse the whole intestinal canal and spur up functional energy, than four times the quantity of any of our ordinary saline aperients. And a continuous use of the same or analogous waters will more thoroughly arouse the liver and dissipate abdominal plethora and its multifarious evil consequences, than any known drugging, with the advantage of inflicting no debilitating injury on the assimilating, blood manufacturing and reparative powers, as is so often done by a too obstinate persistence in the use of mercurial and antimonial preparations. All acquainted with the practice of medicine in the greater part of Continental Europe, will bear me out in this remark; I can not, however, refrain from here quoting the evidence of a well-known English physician and author, Dr. A. B. Granville, who describing his own case, says: "About ten years ago, on passing through the neighborhood of Püllna, (in Bohemia, S. H. S.) heated by a journey of two thousand miles, performed without stoppage by night or by day (no railroads then, S. H. S.); and suffering from that habitual state of the body for which I had, like many more of the inhabitants of London, taken bushels of aperient pills in the preceding years — I was induced on principle, to try the effect of the *Bitterwasser*. The result of that trial was, that I recovered my health—conquered my habitual difficulty of digestion — abandoned all pills — have never taken any since—and find it only necessary to drink about two ounces of the Püllna water to keep all straight."* Another advantage of the aperient mineral waters is that their good effects do not grow less the longer they are used, but on the contrary, with the less need to repeat the dose so often, a smaller and smaller one will often suffice.

In some happy instances, the aperient ingredients are combined with such tonics as iron and magnesia, by which combination, as we all know, and frequently, so knowing, prescribe—the good effects of either are hightened.—Yet still the advantage is on the side of mineral waters, from *the nature of the composition* and the observed superiority of action over ordinary drugs. Thus, that "canny Scot," Dr. James Johnson of *Medico-Chirurgical Review* notoriety, quotes in his "Pilgrimages to the Spas," the following case from his "friend Dr. Heidler," "of a young lady who came to Marienbad (tonic-aperient resolvent, S. H. S.) laboring under sympathetic hectic fever, and who had had hæmoptysis. The stomach would retain no food, especially the dinner. Constipation was obstinate, and the nocturnal perspirations were profuse. The Kreutzbrunn waters were taken, and, after eight days, the fever ceased. In four weeks more the stomach became retentive. Next summer, however, she returned to Marienbad, with the evening vomitings as before. Eight days' course of the water dispelled the sickness, and she recovered her health." I quote this case from a number given in the same work, only because the account of it is so short, and for the present will only trespass on the pages of this journal with one more case from Granville, that of a gentleman whose "long and complicated dyspepsia, hypochondriasm, and weakness of stomach, had been completely cured by a full course of the Kreutzbrunnen." Hufeland used to boast that Goethe had been completely restored to health from a similar complaint by drinking of the same spring. Now, it is but reasonable to suppose, that in these cases, all the ordinary resources of the apothecary had been tried in vain, before expensive journeys were undertaken in search of a health these could not give.

Most remarkable are the effects of those waters containing a predominating

* The Spas of Germany. By A. B. Granville, M. D., F. R. S., etc., etc.

proportion of sodaic salts, especially the carbonate, and which are properly termed alkaline. Nor is this surprising, when we reflect that alkalies are antiplastic and liquefacient, increasing oxydation, softening tissue, dissolving acid matters and weakly organized exudations or deposits, exciting free diuresis, and thus assisting to convey morbid products out of the system. Ordinary diuretics only increase the amount of water excreted by the kidneys; alkalies, by dissolving animal structures, cause the elimination of such organic matters as are the product of a low vitality. Hence the striking effect of these agents in chronic visceral ailments, albuminous deposits in glands, incrustations on the skin, furuncular diseases, old rheumatisms, etc.; and the very considerable proportions of iodine and bromine in the waters of some sources, add to, or advantageously modify, the resolvent powers of the alkaline element.

Best of tonics, *iron*, is of common occurrence, and mostly in that form not easily attainable in every day prescription, although *the* chalybeate, *par excellence*, is the solution of proto-carbonate in water, with excess of carbonic acid. This metal is also quite frequently associated with manganese, now known to possess valuable tonic and cholagogue powers, and to prove curative in anæmic cases where iron alone has failed. No fact is better established, than that blood-diseases in which iron is indicated, yet which have united the administration of the magistral forms of the medicine, have readily yielded to the influence of an appropriate chalybeate mineral water.

Having thus touched on the purgative, resolvent, and tonic properties of mineral waters, viewed as saline solutions, there still remains to be mentioned the important part played by the free gases present in most.

Of the gaseous contents of mineral waters, *carbonic acid* holds the first place, both in frequency of occurrence, and in quantity present—in some instances nearly five volumes — and independent of the important chemical part which it plays as a solvent, its power as a medicinal agent is by no means insignificant. When taken into the stomach in considerable quantities, it lowers the irritability of that organ, while it increases its functional activity, improves the quality of its secretions, and increases its digestive powers. These or similar effects are next produced in the contiguous and related organs — intestines, liver, pancreas, etc.— and while the portal circulation is accelerated, the secretions of the organs named are increased in quantity, and are improved in quality. Then the heart and arterial system feel the benefit of its stimulus, and together with the muscles in general, experience an augmentation of contractile force. Finally, the most distant part of organic life feels its effects, which are shown in improved tone of skin and membranes, more free secretions, and increase of urine. It also checks the tendency to degradation of the fluids and solids, and the formation of pus. It is indicated in all those cases in which acids are generally used, and probably does not produce those injurious effects on the stomach which these occasionally do, because it can not be applied to its surface in so concentrated a form as they can, and too commonly are.

It is deserving of notice, that in latter years carbonic acid in the gaseous form has been extensively used as a bath, especially at Marienbad, Eger, Pyrmont, Meinberg, and some other places, where enormous quantities are perpetually streaming from the surface of mineral springs, or even from holes and fissures in the ground. Its effects, when thus applied to the surface of the body, are similar to those already described as following its internal administration, namely, diminished irritability, increased tone, and improved secretions; and these effects are propagated from the surface to the interior of the body, in the former, just as the reverse takes place in the latter. These baths have proved eminently serviceable in chronic diseases of the skin, including its functional derangements, neuralgia, rheumatism, gout, palsy, amenorrhea, suppressed hemorrhoidal discharges, lymphangitis, etc. Some very remarkable cures have been performed by these baths, in intractable cases of the diseases mentioned above.

*Hydro-sulphuric acid* is far more energetic in its action than carbonic. In large doses, whether inhaled, applied to the skin, injected into the veins, or taken into the stomach in aqueous solution, it first lowers, then suspends, the functional activity of the nervous system—occasions a small, intermitting pulse, general weakness and debility, vertigo, delirium, insensibility, palsy, death. In such small doses as are administered medicinally, however — it rarely nor ever exceeds one and a half per cent. of the volume in a mineral water — its effects are, increased activity of the circulation, especially in the abdominal venous system, rendering the pulse also fuller and more frequent, increasing the secretions of the skin, and of the mucous membranes of the organs of respiration and digestion, and of the urinary apparatus, stimulating the functional activity of the absorbent system, lymphatic glands, and serous membranes. But if too long continued it disturbs digestion, occasions nausea, vomiting, colic, diarrhea, and general cachexia, with a depraved condition of the blood.

In combination with alkalies and alkaline salts, as found in many mineral waters, it constitutes a most powerful agent for the cure or relief of obstinate deep-rooted disorders of the skin, mucous membranes, and lymphatic system, as well as of chronic metallic poisoning, as hydrargyrosis, plumbismus, and such like.

*Nitrogen* occurs free, and in some springs in very large quantities; it appears to be indifferent, or to produce no special curative effects.

Free *oxygen*, occurring less frequently, and in smaller quantities, does not either appear to have any appreciable influence on the remedial power of mineral waters.—*Cincinnati Med. Observer.*

